# Financial risk management strategies of small to medium illicit drug enterprises: considering low-level money laundering

**DOI:** 10.1007/s12117-023-09501-5

**Published:** 2023-09-19

**Authors:** Mark Berry, Mike Salinas, R. V. Gundur

**Affiliations:** 1 Bournemouth University, Poole, UK; 2 Manchester Metropolitan University, Manchester, UK; 3 University of the West of Scotland, Glasgow, UK; 4 Sir Arthur Lewis Institute of Social & Economic Studies (SALISES), The University of the West Indies, Mona, Jamaica

**Keywords:** Drug market, Money laundering, Anti-money laundering (AML), Organized crime, Criminal career turning points

## Abstract

The illicit drug trade generates billions of dollars in revenue per year, much of which comes from wholesale and retail sales late in the supply chain. Yet, the methods retailers and low-level wholesalers use to launder this revenue remain poorly understood. Using in-depth interviews with illicit drug entrepreneurs in the United States and the United Kingdom, this article analyses laundering strategies among such market actors. Our findings indicate that a significant proportion of their illicit proceeds are disposed of through relatively small-scale ‘everyday’ cash transactions (< $1,000) that are effectively untraceable. For those generating more substantial revenues, a variety of accessible and uncomplicated laundering strategies are employed, such as reporting such revenues as taxable income, using proxies to launder funds, and using revenues as investment capital within small-scale legal enterprise. Ultimately, we identify uncomplicated, yet largely effective, methods of laundering criminal proceeds amongst our sample of low- to medium-level illicit drug sellers. Though the sums at an individual level are relatively trivial, the ‘mass of minor offences’ of this nature likely accounts for a significant share of laundered drug revenues in Western consumer drug markets.

## Introduction: the illicit drug trade and the laundering of its proceeds

The illicit drug trade generates significant revenue earned by organized criminal enterprises (Levi et al. 2013; Paoli 2017). Together, the United States and the United Kingdom dominate global consumer spending on illicit drugs. U.S. consumer spending on cocaine, cannabis, heroin, and methamphetamine is estimated to be $150 billion a year (Midgette et al. 2019). The U.K. accounts for one-third of all consumer spending in the European Union drug market (Black 2020), with annual consumer spending on illicit drugs estimated to be $13 billion (£9.4 billion). Though illicit drug supply chains often stretch internationally (Giommoni et al. 2020), the greatest mark-ups and returns on investments occur in the mid- to late-stages of the supply chains in the retail markets of Western Europe and North America (Reuter and Stevens 2007; Salinas 2023). Consequently, significant proceeds of crime are generated at the consumer end of the illicit drug supply chain, and these proceeds must be managed, laundered, and expended. This article examines how these criminal entrepreneurs dispose of these profits and mitigate their financial risks from law enforcement and from competitor offenders using money laundering strategies.

The offence of money laundering occurs whenever revenue from crime is saved or spent, regardless of the amount or its purpose (de Koker 2009). Money laundering is most-commonly conceptualized as the hiding, transfer, or transformation of the proceeds of crime. However, anti-money laundering (AML) regulations, law enforcement agencies, and policymaking and assessment bodies, such as the Financial Action Task Force (FATF), focus on the disposal of relatively large sums of money. To be laundered, dirty money typically requires:

- *placement*, putting the proceeds of crime into the legitimate financial system;
- *layering*, moving those proceeds through the financial system in various transactions to disguise their criminal origins; and.
- *integration*, finalizing reintegration of the proceeds of crime into the licit economy (Levi and Reuter 2006; Unger et al. 2006).

These laundering practices are not exclusive, may overlap, and, when considering small amounts of illicit capital, may not occur at all. Some critique this three-step model as simplistic by failing to account for more complex or varied money flows. According to van Duyne (1998), the term ‘money laundering’ has little explanatory value in illuminating how crime money is managed; this article aims to elucidate this question.

The most common AML regulation requires financial institutions to report “suspicious” transactions, typically defined as payments that eclipse a set dollar amount, usually 15,000 pounds, euros, or dollars, made over a fixed period of time. While AML policies have good intentions, little evidence exists of their effectiveness in disrupting the criminal finances of average criminals (Halliday et al. 2020; Naylor 2002; Pol 2020).

Furthermore, AML policy is designed to both prevent and reduce money laundering. “Follow the money” has become the guiding mantra for law enforcement agencies aiming to disrupt criminals’ financing strategies (Naylor 2002). Anti-money laundering regimes aim to reduce the capacity of illicit entrepreneurs to benefit from the proceeds of their crimes, thus disincentivizing the continuation of a life of crime. In both the United States and United Kingdom, the countries featured in this study, law enforcement agencies are encouraged to target criminal assets and to forensically link illicitly gained money to illicit actors and their illicit networks. Both countries have regimes designed to force criminal actors to forfeit and/or pay back their earnings from crime. Despite these measures, the available data – which are notoriously incomplete – suggest that these efforts have a “less than 0.1% impact on criminal finances” and result in compliance costs that only impact licit actors (Pol 2020, p. 73).

Nonetheless, effective criminal entrepreneurs expand or sustain their illicit businesses over the duration of their careers by laundering or hiding money and/or assets to curb law enforcement’s ability to “follow the money” (Gundur 2020). People who launder money may do so via various roles and strategies, depending on need and access (Naylor 2002). Combining the work of Levi and Soudijn (2020) and Malm and Bichler (2013), we identify four broad categories of launderers.

- *Passive self-launderers* directly launder the proceeds of their crime by purchasing illicit goods that maintain their criminal enterprise, such as additional drug consignments for resale or by repaying debts from previous consignments, or by purchasing licit goods or services, such as groceries, utilities, or expenditure on lifestyle (Levi and Soudijn 2020). The low values and volumes of currency involved mean that passive self-launderers are likely to be neither subjected to significant scrutiny nor included in estimates of money laundering.
- *Active self-launderers* launder their proceeds of crime, typically via legitimate business or investment endeavors. Thus, they may establish licit enterprises or fund undisclosed/off-the-books renovations to business premises (Malm and Bichler 2013). While passive self-launderers simply spend their proceeds, active self-launderers use multistep processes to hide the provenance of funds or dispose of crime money.
- *Opportunistic launderers* launder the proceeds of a crime committed by another person, with whom they have a familial or friendship tie (Malm and Bichler 2013). Drug dealers often recruit these opportunistic launderers from within their social networks. Opportunistic launderers use their own bank accounts or those of friends, families, or local businesses to launder the illicitly gained funds. Like active self-laundering, opportunistic laundering is an active process whereby criminals pursue laundering opportunities present in their immediate surroundings and among their routine contacts (Kleemans 2013).
- *Professional launderers* are enlisted specifically by those with a large surplus of illicitly obtained funds. Professional launderers, rather than being integrated members of any one criminal organization, are often lawyers, bankers, or accountants who provide their services and particular skill sets for a fee (Levi 2021; Levi and Soudijn 2020).

Lost in most analyses of money laundering, and the focus of this study, are the activities of the first three categories of launderers. Notably, the illicit entrepreneurs canvassed within this study do not use professional entrepreneurs. Passive and active self-launderers and opportunistic launderers are the people most likely to be involved in the laundering of the proceeds of crime related to retail or low-level wholesale drug sales. Supplying illicit drugs rarely funds opulent lifestyles; monthly earnings among retailers might total a few hundred dollars; others may earn up to several thousand dollars per month (Salinas 2023). Thus, some retail or low-level wholesale illicit entrepreneurs may develop a steady cash flow that needs laundering to be easily spent. This study confirms previous findings in the U.S. and the U.K.: those who engage in small-value and small-volume laundering efforts are not particularly sophisticated and often engage in opportunistic strategies which rely on their proximal networks of friends and family members (Levi and Reuter 2006; Malm and Bichler 2013; Naylor 2002; Ouellet et al. 2016).

Beyond this confirmation, this article makes five contributions to the existing literature. First, it describes capital-management strategies used by illicit entrepreneurs who retail and engage in low-level drug wholesaling. Second, it shows that illicit entrepreneurs who seek to make their illicit businesses their primary source of income and those who seek to exit illicit business and establish licit businesses engage in similar laundering practices. Third, it notes that clear business “turning points” exist, regarding the needs and strategies of laundering proceeds from drug sales (Carlsson 2012; Gundur 2020). Fourth, it documents how illicit entrepreneurs innovate by deploying new and emerging laundering opportunities. Finally, it describes how some illicit entrepreneurs use licit financial structures to legitimate the proceeds of illicit businesses.

This article proceeds by presenting the current study and its methodology. Then, it provides an analysis of the financial considerations and strategies practiced within the various business contexts. Last, it discusses the implications these strategies have for law enforcement and policy.

## Current study and methodology

A limited number of empirical studies have detailed the financial strategies used by low-, mid-, and upper-level illicit entrepreneurs (Gundur 2020; Malm and Bichler 2013; Naylor 2002). Moreover, little qualitative research examines the practices of such entrepreneurs, particularly regarding innovative uses of proceeds of crime that sustain and expand small-to-medium illicit enterprises or help illicit entrepreneurs transition to the licit economy. Accordingly, we pose three research questions:

1. How does one’s position in an illicit entrepreneurial career impact one’s choice of money management strategies?
2. How do illicit entrepreneurs use licit pathways and structures to launder their proceeds?
3. Why and how do illicit entrepreneurs innovate to launder the proceeds of their crimes?

This article draws on three ethnographies that involved retailers and wholesalers of illicit drugs. One occurred primarily in the United States and two occurred primarily in the United Kingdom. All three studies collected the bulk of their data from major urban centers. This article draws on a subset of the interviews undertaken in these ethnographies. This subset includes 84 semi-structured, face-to-face interviews, of which 13 were with members of law enforcement and 71 with people who were previously or currently involved in the drug trade and spoke of illicit finances. Of the interviews with criminal entrepreneurs, 23 (7 in the U.S. and 16 in the U.K.) were in “upper-level” positions within their illicit enterprises; these included illegal drug suppliers who engaged in wholesale distribution or ‘managed’ teams/networks of suppliers, as well as “specialists” who engaged in specific activities, such as weapons trafficking or money laundering.

The data presented in this article derives from formal audio-recorded interviews, which lasted from one to three hours; it excludes informal conversations that were recorded via fieldnotes. Most participants were interviewed formally only once; some were interviewed further in formal or informal settings. Most interviews were audio recorded and then transcribed; quotes are from these transcriptions. A minority of respondents preferred not to be recorded; these interviews were recorded in written notes. The interview data was analyzed using a grounded approach.

The base interview schedules for all three studies focused on the motives and mechanics of retail and low-to mid-level wholesale illegal drug suppliers and covered similar content. To protect the identities of the studies’ participants, pseudonyms are used throughout in reference to people and places. All the individuals quoted within this article are specific individuals; there are no composite characters. The three ethnographies, used in this article, were approved by the relevant university ethics committees.

Participants were recruited using three recruitment methods: traditional chain referral/snowball methods; online classified advertisements (Gundur 2019b; Worthen 2013); and direct engagement and “gonzo” recruitment, which took advantage of the authors’ social and life circumstances to contact and interact with participants (Innes 2014). The diversity of recruitment methods allowed accounts to be collected from individuals who were unknown to one another and whose businesses operated independently of other participants. This strategy allowed the authors to identify common patterns of entrepreneurial behavior between cohorts. A preliminary analysis of the data revealed that launderers use similar tactics regardless of their location. Consequently, a geographically comparative approach was not employed.

## Limitations

The nature of the sample limits the generalizability of the arguments made in this study. It is not possible to gain a representative sample of active and retired criminals from the community setting because their true number is unknown. Certainly, the nature of the behaviors examined in this study makes survey methods difficult to execute; not studies to our knowledge have undertaken such an endeavor.

Moreover, the interview schedules differed among the cohorts, and, given that the interviews were semi-structured, different topics emerged across offender narratives. The relative levels of the respondents and their comparative access to the markets they traded in varied considerably. Nonetheless, insight into the activities of these actors is rare and illuminates practices that differed in comparison to studies which drew upon ‘failed’ offenders, that is successfully apprehended and prosecuted people, incarcerated in the prison system (Hobbs 2013). Much of what is known about the motives and mechanics of drug supply amongst this subpopulation, including their financial strategies, stems mainly from interviews with incarcerated offenders (e.g., Decker and Chapman 2008; Desroches 2005; Matrix Knowledge Group 2007; Reuter and Haaga 1989). The questions raised in this research could be the basis of further studies on money laundering behaviors, which specifically focus on the that issue rather than examine it as a function of a larger program of study.

Though there is substantial merit in the methods and samples of the studies cited above, some argue vehemently that active offenders must be studied in the free world, where they live and operate, rather than in the confines of an institution, to fully understand their motivations and means of operating (e.g., Jacobs 2000; Wright and Decker 1994). Accordingly, this study adds to the body of ethnographic work on active illicit entrepreneurs (Adler 1993; Grundetjern and Sandberg 2012; Hobbs 2013; Jacques and Wright 2015; Moyle 2019; Sales and Murphy 2012), by focusing specifically on financial aspects, which are frequently neglected despite their inherent policy importance vis-à-vis money laundering regulations.

The following section begins with an analysis of small-scale passive self-launderers before demonstrating how laundering strategies become more sophisticated as illicit enterprises grow in size. It then considers how criminal entrepreneurs shift to less risky practices or legitimize their operations to ensure long term financial sustainability.

## The financial considerations of small- to medium-scale illicit entrepreneurs

The drug trade consists of an unquantifiable number of loosely-structured illicit entrepreneurs who work in concert to distribute products across a global supply chain (Purvis and Gundur 2019). To understand how financial implications depend upon economic context, it is helpful to classify illicit enterprises based on their size and corresponding financial turnover. While large hierarchical enterprises have been documented within the drug trade (Padilla 1992; Venkatesh 2006), most retail and low-level wholesale drug businesses are relatively small (Densley et al. 2018; Gundur 2019a). This study demonstrates that financial considerations are context dependent and vary greatly depending on the volume of money transacted. Thus, while some criminal entrepreneurs launder funds to expand or maintain their illicit enterprise, others design their economic strategies to exit illicit enterprise altogether. Moreover, although illicit entrepreneurs generally employ the simplest available strategies to use the proceeds of their crime, some may innovate when standard practices are no longer viable or effective.

### Small-scale illicit entrepreneurs and passive self-laundering

People start dealing drugs for many reasons. Some start as children. American gang old-head Renzo ran kids on the street corners to retail drugs. The $50 per day the children could earn at age 7 or 8 was more money than they could imagine. Some, like American gang member Daryl, sought “to make a little extra money and support a [drug] habit.” Others, like American gang member Wilfred, was a full-time student who sold on his university campus to have an auxiliary income. Similarly, English drug dealer Ryan, who worked in a takeout chicken restaurant, turned to drug dealing as a means to supplement a source of licit income (Salinas 2018). And some illicit entrepreneurs, such as English nitrous oxide distributor Max, aspired to expand and grow their trade into a business that would become their primary source of income.

No matter the individual strategies or objectives, involvement in the retail drug trade often began at the bottom of a supply hierarchy or, at least, in positions with low financial stakes. Within this study, 67 actors reported starting their careers at a lower level. As with licit entrepreneurs, illicit entrepreneurs are constrained by the available opportunities within their immediate network as well as limited liquidity or credit (Satterthwaite 2012; Torres et al. 2020). Opportunities come in various forms, including *inter alia*, being embedded within social networks of recreational or intensive drug users; being affiliated with a street gang engaged in retail drug sales; or being a salaried employee in a drug processing business. Most of these entry-level jobs entail high risks for low rewards; however, some risks can be mitigated by dealing exclusively to one’s community (Gundur 2022; Jacques and Wright 2015).

The revenues were typically first reinvested into the drug trade, to either sustain or build the retail business, with remaining net profits expended elsewhere. “I’d always separate my re-up money [that is, money to resupply inventory] from my profit money,” Linus, an American cocaine retailer, explained. “My re-up money was first. So, when I made my re-up money, I went and bought me another [eighth of an ounce of cocaine to break down and resale]; and then my profit money came last.”

Net profits were commonly used to underwrite day-to-day expenses, such as rent, utilities, groceries and transportation. Such practices were commonplace across the three cohorts of this study and mirror patterns of behavior identified in the empirical literature (Fader 2019; Nguyen et al. 2023; Vannostrand and Tewksbury 1999). “[Selling cocaine] enables me to run my car, to see my daughter, to put food in the cupboard. So that’s the main things, really,” explained Ryan. “I can’t even say it’s enough to cover to buy clothes or things like that. I save to buy TV, internet.” For other, more profitable entrepreneurs, or for those facing a relative windfall in revenues, drug revenues were frequently expended via cash purchases of luxury consumer goods and services, from designer clothing (such as a $420/£350 winter coat by *G-Star*, or a $150/£120 woolen hat by *Prada*) to dining in restaurants (e.g., a $150/£120 bill for food and drinks).

These mundane, quotidian transactions are, by definition, money laundering and constitute *passive self-laundering* activities: no active laundering strategy is needed to make the earnings expendable. The relatively small value of these cash transactions – typically less than 50 dollars or pounds per transaction, and rarely totaling more than 150 dollars or pounds per transaction per week, per supplier – resulted in untraceable purchases. Accordingly, this money purchased everyday items while underwriting a lifestyle furnished with some consumer luxuries. This purchasing activity seemingly did not raise suspicion, even though aggregate monthly spending on such items totaled several hundreds or, in some cases, thousands of dollars or pounds. Moreover, for those living especially economically precarious lives, little, if any, proceeds of crime were saved or deposited in formal banking institutions. Thus, no auditable record was created, making it difficult for law enforcement to readily identify, punish, and deter such behavior. This difficulty explains, in part, the limited state expenditure directed toward policing these behaviors (Torres et al. 2020).

Respondents who had both licit and illicit incomes typically saved a proportion of their legal revenue. These people deposited their legitimate incomes into their bank accounts and used their illicit proceeds for their day-to-day necessities (see: Nguyen et al. 2023). Over time, the licit funds in their bank accounts were used for larger, legitimate purchases. Tee, a full-time office administrator, who supplemented his salary with cannabis sales to his friendship networks, engaged in such behavior. “I try putting as little as possible though on my [bank] card and really I’m just spending my weed money,” explained Tee. “I’ve a few direct debits coming out [of my bank account each month,] but mainly the money I’m getting from [my salary] is just building-up […] for a deposit for a house.”

Overall, passive self-laundering proved adequate for drug sellers with nominal to modest illegal cash revenues. It also allowed some drug sellers to save a larger proportion of their licit revenue, which could be unproblematically used to make substantial, traceable purchases. Weekly illicit cash revenues equivalent to several hundred dollars or pounds could be spent easily via everyday expenditures and luxury consumer goods and services. However, once illicit revenues outpaced such expenditures, illicit actors required more deliberate, active means of laundering revenues or accrued savings to reduce risks of detection and to sustain involvement in the drug trade. Our findings indicate that when annual profits exceed some few tens of thousands of dollars or pounds, illicit entrepreneurs recognize the need to manage their proceeds of crime. Within our studies, 16 of our 23 upper-level participants reported having profits within this range.

### Active self-laundering among medium scale traffickers and suppliers

The *expansion* phase in illicit entrepreneurial careers typically involves expanding business and increasing revenue. Expansion efforts vary and may involve retailing more product, entering the wholesale trade, or diversifying into new illicit markets. Successful expansion often results in income beyond what is easily disposed via passive self-laundering. Illicit expansion, however, is not a singular goal; as we discuss later, some illicit entrepreneurs expand their illicit enterprise to generate increased financial capital necessary to transition towards legal enterprise.

Once illicit entrepreneurs achieve the revenue needed to optimize their reward-to-risk ratio, some stop expanding and maintain stasis within a “sweet spot;” continued expansion could expose them to increased and intolerable risk (Fader 2016; Gundur 2020). Others simply lack the economic and/or social resources to expand further. Accordingly, *maintenance* represents sustained criminality and requires illicit entrepreneurs to develop strategies to actively launder and spend their proceeds of crime without attracting attention from law enforcement.

Of the 23 illicit entrepreneurs in upper-level positions within their enterprises, 19 had engaged in *expansion* and, later, *maintenance*. For example, Renzo first retailed drugs as a gang member; later, he became the go-to wholesaler for his gang. Similarly, Teeth went from retailing individual hits of heroin with a friend to running a crew of drug retailers. In our sample, all the upper-level drug dealers expanded their illicit businesses in similar ways. Likewise, most maintained their expanded businesses for a protracted period, firmly establishing themselves in an economic sweet spot (Gundur 2020). In this position, these illicit entrepreneurs continued to operate their criminal enterprises; they found the risks inherent to their illicit trades to be acceptable, given that they had earning potential as good as, or better than, successful white-collar professionals. This income not only allowed these entrepreneurs to reinvest into their businesses, but also provided sustained and significant profits, which required laundering to become expendable. The actors who disclosed their income reported revenues between £/$250–1050 per week depending on the product sold (i.e., powdered cocaine was more lucrative than cannabis), with those in upper-level positions earning towards the top end of this scale. The illicit entrepreneurs predominately used two laundering strategies: active self-laundering through false pretexts and opportunistic laundering through proximal relationships.

### Active self-laundering through false pretexts among mid-level traffickers and suppliers

Active self-laundering is a process through which illicit entrepreneurs launder the proceeds of their own crimes, typically through legitimate businesses. Although money laundering is often associated with tax evasion in official and some academic discourses (Levi and Reuter 2^006^; Maugeri 2018; Unger 2013), our data indicate that illicit entrepreneurs who self-launder often reported their criminal proceeds as legitimate income to tax authorities, thus rendering it taxable income (Naylor 2002). In this study, 9 respondents owned a legitimate business; 5 reported illicit earnings to the tax authorities via activities, such as feigned sales, clients, expenses, or hours worked.

By fraudulently disclosing illicit income as legal income, and paying the associated taxes, these illicit entrepreneurs created a plausible backstory to their income while reducing their chances of being investigated by the tax authorities or law enforcement. Law enforcement, in the past, has at least sometimes used the tax code to bring proceedings against prominent and often elusive criminals, with Al Capone perhaps the most infamous example (Bucy 1997). Nonetheless, regardless of jurisdiction, “tax agencies do not profit from and are not set up to investigate overreporting of taxable income” (Levi and Reuter 2006, p. 354). Thus, paying taxes on illicit income is a simple and common way of self-laundering; one can overreport revenue and pay taxes on it, rendering the reported money clean (Naylor 2002). Moreover, tax authorities are unlikely to review the tax returns submitted by the illicit entrepreneurs in our study; the revenue and expenses claimed are below the £/$15,000 single payment thresholds that would typically require a review. Simply put, where tax authorities are concerned, capacity is low; public sector cuts have resulted in declining review, investigation, and enforcement actions undertaken by U.S. and U.K. tax authorities (Internal Revenue Service 2019; Lobao et al. 2018). Nonetheless, laundering money in this manner entails costs; taxation can be expensive.

A traditional strategy of self-laundering involves investing in or operating licit businesses, including off-licences/liquor stores, food outlets, bars, nightclubs, hotels, money exchanges, barber shops, maid services, real estate purchases, and arms trading (Buchanan 2004; Grosse 2001; He 2010; Levi and Reuter 2006). Pancho, an American prison gang member who retailed drugs, talked about investment within his community and how gang members who had extra money would “get people that ain’t involved, people who’re legit” to start a new business “from scratch, little by little.”

Another prison gang member, Bruce, talked about how the well-established higher-ups of the gang created businesses to launder their drug money. “[The higher-ups in the gang] got smart, where they own construction businesses, restaurants, where it’s easy to wash that money,” Bruce explained. “They know that they can launder that money. There are large amounts of money that you can lose in construction or large amounts of money that you can make in construction. And you don’t got to report everything. You could [claim] it as a loss, but you got cash.” Fraudulent expenses can reduce revenues on paper but do not reduce real cash available, thus providing laundering opportunities.

Other attempts to launder money through investments showed that a successful illicit business does not guarantee a successful licit business or a sustained avenue for laundering money. In addition to his salary from full-time legal employment, Sol earned weekly net profits of around £3,000 ($4,100) from wholesaling cocaine consignments. Initially, Sol passively laundered his illicit income through ordinary spending and saved his licit income. His savings quickly increased; with an excess of cash, Sol began using the funds as investment capital:

I’ve got my wage coming in […] so it’s not an issue paying five-grand here-and-there to get [business leases etc.… But as] they’re all cash businesses […] once they’re up and running I’m able to put me own [criminal] money into them, no issue. […] The [pub’s] whole refurb was paid for cash [off-the-books.…] Same with [the other businesses]. Loads of people I know are happy to work for cash – works out better for them [tax-wise].

Sol’s partner in his pub business was cocaine retailer Cliff. Despite Sol’s positive outlook on his investment, the business faltered, and Cliff admitted that the pub business was a failure that cost them what they had invested.

Some illicit entrepreneurs used their licit businesses to report their illicit earnings as legitimate ones to the tax authorities. A prime example is Charley, an Englishman who maintained his previous, legitimate work identity to provide cover for his illicit earnings: “I’m currently paying money into the bank as a self-employed electrician.” Charley created invoices with “different names, different addresses, different amounts,” to account for the income he reported, while manipulating expenses: “I put minimal receipts in, so, the jobs I do give to people who I pass my work onto, they do provide me with the receipts for their material.”

Initially, Charley’s fulltime salary, paid to him by one Chinese and two English ‘bosses’ who ran the illicit image and performance enhancing drugs (IPEDS) business, netted Charley some £1,200 ($1,650) per week, about double the U.K.’s average weekly wage in 2020. As his illicit enterprise became more efficient, Charley’s income increased significantly. Throughout this time, he used his electrician business to report a portion of his illicit earnings.

Charley went on to say that the IPEDS business grossed around £1,000,000 ($1,376,000) in revenue per year. The net profits were split among the three managers who had their own fronts, such as tanning and nail salons, through which they reported earnings to the tax authorities: “At their level they are happy to pay the tax bill. They are happy to pay [a] four-grand-a-year, five-grand-a-year tax bill. But then it benefits them, ‘cause the more they [pay in taxes, the more they can borrow] from the banks.” Charley’s managers gladly paid income and business taxes since doing so allowed them to develop good credit ratings. A £5,000 ($6,900) per year tax bill equates to reported earnings of around £37,500 ($51,500) per year, a reasonable income for a small tanning or nail salon.

Reporting greater amounts, however, can become costly. For example, Rushton’s occupation as a self-employed personal fitness trainer enabled him to launder illegal revenues via bogus clients and training sessions. Rushton netted monthly revenue of around £3,000 ($4,100) through his brokerage position in several drug supply networks. He reported surplus, illicit income alongside his legitimate monthly earnings of approximately £1,500 ($2,050) to the U.K. tax authorities (HMRC). The inclusion of these additional funds pushed Rushton into a higher tax bracket and led him to pay several thousand pounds in additional income tax and national insurance contributions. Approximately one-quarter of his £36,000 ($49,500) criminal revenue was spent laundering this money. Thus, illicit enterprises may restrict their size because increasing operational scale increases not only risks and complexity (Edwards and Levi 2008), but also financial costs. Economies of scale do not benefit illicit enterprises in the same manner as licit enterprises. Laundering the proceeds of crime by declaring them as legal income can incur a heavy financial penalty. Nonetheless, this process launders money in a predictable manner.

Max supplied nitrous oxide, a psychoactive drug that straddled legal boundaries insofar as it, at the time, could be sold/supplied legally for use within industry (e.g. the culinary sector) but cannot be sold/supplied when use relates to its psychoactive properties. Max reported all his business revenues and expenses to the tax office. “Everything is above board; I have an accountant who does all my taxes,” Max explained. “Because it’s legitimate business, I can claim back my mileage; I get 45 pence a mile. I don’t always claim because I don’t always keep track of it, but I try and claim for most of it.” After expenses, Max was reporting profits of £100,000 to £150,000 ($137,500 to $206,000) per year to the tax authorities, rendering the proceeds laundered. Max could save or spend that money as he chose; however, had the police investigated his nitrous oxide business and prosecuted him for selling it for recreational use, having paid tax would not have protected these sums from confiscation.

In brief, illicit entrepreneurs understand the benefit of declaring their criminal revenue for tax purposes and develop methods to report their illicit proceeds of crime as legitimate earnings, particularly when there are few resources to investigate their businesses. The illicit entrepreneurs at this level prefer reporting their income via such means to developing laundering strategies that rely on others. Relatively high amounts of illicit earnings can be laundered this way, although at a significant cost. Moreover, when individuals cultivated a licit identity to front illicit behavior, some displayed assets, such as cars and houses, which accorded largely with their front identity. Thus, paying taxes allowed illicit entrepreneurs to blend into the licit economy and mainstream society. Nevertheless, some illicit entrepreneurs were not interested in, or could not fully depend upon, this strategy; consequently, they laundered their money via other people or entities.

### Opportunistic laundering via proximal relationships

Not all illicit entrepreneurs who have significant illicit earnings seek to legitimate their illicit earnings by reporting them to the tax authorities or investing in licit business. In large illicit enterprises, professional launderers, who launder large quantities of illicitly earned money consistently, are part of the business model. Small to medium scale illicit entrepreneurs, even if they are in the upper-echelons of their illicit enterprise, however, typically do not have access to professional launderers. Instead, when they have more proceeds than they can self-launder, some, including 8 of the 23 upper-level actors in our sample, seek laundering opportunities via their proximal relationships. Using opportunistic launderers is an active strategy whereby drug dealers pursue opportunities among their routine contacts (Kleemans 2013). By displacing the laundering operation to a proximate party, the illicit entrepreneur creates a “cutout” or a buffer that diminishes the risk of being caught for illicit activity (Gundur 2020; Levi 2020). This activity shields the criminal entrepreneur from the purview of the authorities. “I don’t keep anything in my name,” Teeth explained. “I sublet. I’m not on the voters list. Even the social [the Department for Work and Pensions] don’t know where I live. I’ve put myself down as homeless.”

Nonetheless, Teeth wanted to buy expensive things, like a sports car. If he purchased the vehicle outright, he would likely draw unwanted attention from the police. Consequently, Teeth conducted a “strawman purchase” (Teichmann 2020) via his taxi driver brother; the car was purchased and insured in his brother’s name. Teeth also avoided driving the car in the city to reduce police attention, allowing him to blend “into the flows of city life” (Berry 2018, p. p5). Similarly, Renzo bought a house and put it in the names of his ex-wife and adult children, who had legal jobs. Damián, a former weapons dealer, spoke of a similar strawman purchasing process: “If I wanted to buy a new car, I wouldn’t go to the car dealership. I would get one of my friends that had his own business or a working man, and I said, ‘Buy this for me.’ And they would buy it for me. And I would pay them a little extra.”

With strawman purchases, illicit entrepreneurs generally relied on a relatively small network of facilitators, prioritizing kinship and group identity in their recruitment (Gambetta 1990; Malm and Bichler 2013). For instance, American gang member Lil G explained that some in his gang would recruit “a cousin of a friend or a family member” who worked legitimate jobs and who was positioned to engage in ‘placement and layering’ processes. Charley laundered his salary payments from his mail-order anabolic steroid sales via “money mules” he recruited from his social network. Charley paid his mules a fee to receive structured payments through Western Union – always below the mandatory reporting threshold that would trigger a suspicious transaction report – and then withdraw the cash. Charley noted that recruitment was easy in Britain where austerity, economic instability, and rising levels of unemployment created an economic situation where, for many, the opportunity to make fast money was hard to turn down, especially while legal consequences seemed improbable (Arevalo 2015). Charley’s pool of money mules was large enough that he could use any given individual intermittently, reducing the chance an investigator would notice a pattern.

In short, illicit entrepreneurs pursue laundering opportunities that have the least complexity and resistance. However, when these opportunities are not present or easy to access, innovation becomes necessary.

### Innovation

Innovation has long been a strategy used by people who seek better opportunities than those presently available (Merton 1968). In illicit finance, innovation involves improved strategies for risk avoidance or risk transfer (Fader 2016). In our sample, 5 of the 23 upper-level respondents engaged in innovative tactics to respond to fluctuations in access to laundering opportunities and shifts in the use of cash and other payment mechanisms. In 3 cases, proximal arrangements went beyond friendly, collaborative arrangements towards threatening, coercive ones. Damián described how drug trafficking organizations would racketeer legitimate business owners from their own ethnic communities. Instead of demanding payment, the drug trafficking organizations demanded that the businesses laundered dirty money: “[The drug trafficking organizations] would threaten the business owners. If they didn’t do what they wanted them to do – let them wash their money through there, let them write receipts for such and such – you know, they’d shut them down.” Thus, the drug trafficking organizations could launder large amounts of money without owning businesses.

Three of the upper-level actors used trade-based strategies or alternative payment systems to transact value and minimize their use of cash. Damián, for instance, transacted high value for drugs. He avoided dirty money at the lower echelons of the drug trade by trading firearms for drugs. He acquired firearms from criminal associates or from gun shows at good rates and traded them for an inflated value to the drug wholesalers. He then used the proceeds from the drug sales to pay for more firearms which he traded or resold. Charley’s anabolic steroid enterprise also expanded its payment options by accepting the cryptocurrency Bitcoin, fiat currency via Western Union, and anonymous tokens purchased by credit card; all of these are difficult (though not impossible) to trace.

The shift away from cash in the licit economy also marks a need to innovate in the illicit economy. The use of cash as a payment method in licit transactions has declined significantly over the last decade, with payment cards being the most common form of payment in the U.K. and U.S. (Kumar and O’Brien 2019, p. 3; UK Finance 2020, p. 2). Policymakers and law enforcement see the decline of cash as a benefit; cash facilitates predicate crimes, money laundering, and corruption (Riccardi and Levi 2018; Schneider 2017; Singh and Bhattacharya 2017). With cash less commonly available for retail sales of drugs, Teeth adapted by offering his clients and staff members drugs on credit. To ensure payment, Teeth had his clients give him their debit cards and pin numbers, thereby gaining full control over their bank accounts and the deposits, which were mostly government benefit payments, made to these accounts. This process served three purposes. First, Teeth could (almost) guarantee his revenue. Second, Teeth could spend money without having a digital paper trail connected to his name, recreating the anonymity of cash transactions. Third, Teeth could use ordinary payment systems, affording him the ability to further control and exploit his clients, which is critical to sustaining his business in an invisible manner that subverts state surveillance (Berry 2018, 2019). Nonetheless, these opportunities may be less available in drug markets that have fewer ‘intensive users,’ addictive products, and exploitable clientele.

### Risk diminishers, legitimators, and leavers: transition within and exit from illicit enterprise

Within the most prominent form of illicit entrepreneurship – the trafficking and distributing of illicit drugs – offending careers are generally time-limited, sporadic, and often used to supplement, rather than wholly displace, legal forms of work (Fader 2019; Reuter et al. 1990; Salinas 2023). Over time, and through the accrual of stakes in pro-social institutions and communities via licit work, business, and family, many offenders reassess the risks of sustained criminality and seek to exit from behaviors that carry risks they are no longer willing to tolerate (Carlsson 2012; Gundur 2020). Thus, many successful illicit entrepreneurs enter a transitional phase in their criminal entrepreneurial careers, whereby they transform their illicit enterprises to diminish risk, legitimate their businesses, or exit self-proprietary business entirely to take on other legitimate work.

### Risk diminishers

*Risk diminishers* sustain their involvement in illegal economic activities, while shifting towards activities seen to pose fewer or less significant legal risks. Those who seek to diminish risk, but not exit, do so by engaging in activities that are less visible or that breach fewer laws with lower punishments. For example, entrepreneurs in the drug trade may shift their position within the supply chain to reduce exposure to economic risk and/or risk of capture, by moving from retail to wholesale (Gundur 2020). Other entrepreneurs may shift towards products that are less risky to sell, due to their legal status, such as unregulated, decriminalized, products, like “legal highs” and IPEDs; or their low enforcement priority, such as cannabis; or whose misuse is “off-label” abuse, such as the unauthorized consumption of Ritalin (methylphenidate), Adderall (amphetamine/dextroamphetamine salt mixture), or Oxycodone (see, for example: Brewster 2018; Lucas 2018; Rewbury et al. 2017; Salinas et al. 2019).

Of the 71 drug-involved people in our study, 16 reported successfully diminishing risk. Although most were aware of risk, they had varying assessments and appetites for it. For example, Bryan, an American prison gang member, diminished his risk by starting a boutique cannabis business, producing “wax,” a concentrated preparation of cannabis essential oil. He sold to university students and viewed his business venture as less risky than the methamphetamines his gang traditionally traded in: “You start slinging it to [a hip university student], he slings it to his friends; and then you just kick back and don’t do shit. Make wax and kick it. There’s no risk because how many potheads have ever told on anybody? No potheads going to tell on anybody because it’s not a crime. You know what I’m saying? In this place, it’s kids. I don’t know. See, it’s pretty high yield, low risk.” Although cannabis distribution in the area where Bryan lived was illegal at the time, Bryan understood that the prevailing attitudes of the town meant that law enforcement would expend little effort to chase him or his clients.

### Legitimators

*Legitimators* reposition their illicit businesses towards legitimate trade along what organized crime scholar Dwight C. Smith, Jr. (1980) termed the “Spectrum of Enterprise,” with fully licit and illicit enterprise marking each pole of that spectrum. Legitimation attempts may be characterized by a change in products traded or the formal establishment of a business that reports its revenues to the tax authorities. These businesses engage in partially illicit or semi-legitimate trade that generates clean, spendable proceeds that are reported as revenue from their legal business activities.

Within this study, 4 of the 23 upper-level illicit entrepreneurs reduced risk by becoming legitimators. Max’s entrepreneurial evolution is an example of a legitimation shift. Max’s first foray into illicit enterprise involved selling cocaine. As a cocaine dealer, Max saved his excess cash by “putting money to the side.” The profit he gained from dealing provided him the economic foundation necessary to transition from the retail sale of cocaine, an illicit substance, to nitrous oxide, which, at that time, was an unregulated “legal high” in the United Kingdom, commonly used by recreational users (Randhawa and Bodenham 2016). It was legal to sell nitrous oxide, so long as it was not for human consumption. The startup costs for his business were around £5,000 ($6,800) and involved buying nitrous oxide and creating a website where customers could place their orders. Business start-up cash infusions are common in the licit world and are not typically scrutinized by tax authorities. The shift to selling nitrous oxide was not a complete exit from offending behavior; despite the disclaimer on his website, Max knew his customers were consuming the drug. Max managed his finances transparently, declared the proceeds of his business through the tax system, and operated his business as an entirely legal entity, which differentiates him from the risk diminishers. Notably, when nitrous oxide became an unambiguous illegal high, Max abandoned this business altogether.

Similarly, Khalid transitioned from cocaine drug supply to a fully licit, retail electronics store in a busy commercial district. Nonetheless, Khalid persisted in illicit behavior; he underreported his income to the tax authorities by excluding revenue earned through cash sales. “My legal risk [now] is minute compared to the drugs,” explained Khalid. “[The tax authorities would] probably hit me with a fine for not declaring it [if caught], but that’s better than being caught selling coke.”

### Leavers

*Leavers* are those who stop engaging in illicit enterprise and criminal activity altogether. Of the 71 drug-involved people in this study, 45 reported that they had stopped offending. The motivations for cessation varied. Many experienced common turning points that stop offending behavior, including newly emergent legitimate income opportunities, parenthood, incarceration, a near-death experience, or the loss of a peer through incarceration or death (Carlsson 2012; Gundur 2020; Melde and Esbensen 2011). Strategies for leaving illicit enterprise behind also varied, but typically relied upon having a legitimate revenue source sufficient to displace their reliance upon illegal earnings.

Several individuals transitioned towards legal entrepreneurship. Often, though not always, these businesses began as money laundering vehicles. When no longer requiring infusions of dirty money to stay afloat, these businesses became full-fledged legitimate endeavors. After retailing cannabis for several years, Abu invested much of the proceeds into his father’s ailing food production business. His investment modernized the industrial kitchen, which improved business productivity. However, his father’s business ultimately required Abu to invest not only his finances but also his time, which precipitated a sustained exit from the cannabis market.

In other cases, licit businesses were established using criminal finance with the explicit purpose of providing a livelihood that would facilitate criminal desistance and a means to exit offending. American drug distributor Biggs invested proceeds from cocaine wholesaling into his music studio, which allowed him to abandon his involvement in the drug trade, eliminate his risk of being incarcerated, and pursue his passion. When shifts in legislation posed legal risks, Max took his earnings from his nitrous oxide business to start a fully legitimate and legal alcohol delivery service, while also training to become a licensed taxi driver.

U.S. law enforcement personnel indicated that using the proceeds of crime to invest in legitimate business mirrored legitimate cash infusions. Consequently, it was difficult to determine whether new businesses were being financed by or were facilitating the laundering of the proceeds of crime, or whether they were, in fact, legitimate businesses started without illicit money. “It’s very hard for us to distinguish [dirty money laundering fronts from legitimate businesses],” Odalis, an American police sergeant, explained. “It may be a very honest looking restaurant operating on its face, generating good revenue; you see people eating there on a daily basis. But on the back burner, they may be involved in money laundering as well. We would have to have the right people in place, the right informants, to be able to identify these businesses. So, on face value, they’re very, very hard to identify.” A similar reality has been reported in the U.K. (Levi 1997).

Other leavers simply pursued or sustained legitimate employment as a means of transitioning from crime. The U.S. gang member, Renzo, diminished his risks not only by engaging in wholesales, but also by engaging in legitimate employment that allowed him to live a seemingly normal life: “I go to work. Obviously, I got a job now. I work, come back, sit here.” Wrangler, an American who spent time in prison for burglary and drug supply, left offending because the risk outweighed the reward. “I can make $10,000 in ten minutes [through crime], but how much risk do you have?” Wrangler pondered. “I’ve tried to think, ‘So if I got popped with that one pound of meth, that’s approximately ten- or fifteen-years’ sentence for me probably. So, even making $8 an hour, you’re going to make about $20,000 or $30,000 a year or whatever you make. About $24,000 maybe. Think about 10 years at $24,000: That’s over $200,000 that I would lose trying to risk that money. And those odds aren’t great for me anymore.”

The revenues generated via illegal entrepreneurship often exceeded those of legal endeavors. Nonetheless, licit income does not need to match or exceed illicit earnings to trigger cessation from illicit enterprise; it does, however “need to allow for more than mere subsistence level living” (Salinas 2023, p. 10). For many leavers, incomes from legitimate entrepreneurship or employment were often “side bets,” but as legitimate occupational or entrepreneurial returns increased, the comparative or ongoing risks of the drug trade made exit increasingly attractive (Salinas 2023). Investing in the legitimate economy gave these offenders a stake in conformity whether or not they had originally intended to go straight (Sampson and Laub 2017).

Of the 23 upper-level illicit entrepreneurs in our study, 3 made no effort to diminish risk, legitimate their business practices, or leave offending. Of the remaining, all engaged in some form of desistance: 4 were risk diminishers; 13 became leavers, including all 4 of the legitimators; 2 appeared to be in the process of transitioning out of offending; and 1 surrendered to law enforcement. Accordingly, when licit opportunities present themselves, many entrepreneurs engaging in criminal business activities take those opportunities.

## Discussion: lessons learned and implications for law enforcement and policymakers

Policymakers and investigators predominantly consider money laundering in the context of large-scale, professional laundering mechanisms. A 2020 Home Office review of illicit drugs in the U.K. stated that: “Organised Crime Groups will typically launder the proceeds of crime through a variety of means, including cross-border movement of physical cash” and called for greater investment in the policing of laundering of drug money (Black 2020, p. 14). Yet, in absolute terms, much of the money generated from consumer sales remains at the retail-end of the supply chain. Thus, knowing how illicit entrepreneurs engage in money laundering domestically, and which of their offences are likely to draw law enforcement response, is important.

This study has provided an analysis of common laundering mechanisms employed by small and medium illicit drug entrepreneurs across various business contexts. By drawing on three cohorts of respondents, we have been able to identify similarities in money laundering strategies used by mid-to-upper-level illicit entrepreneurs in each country. The U.S. and U.K. are two of the main consumer markets for illegal psychoactive drugs and have similar thresholds, regarding what is flagged as money laundering activity. Country-specific legal and enforcement contexts did not seem relevant in understanding the offenders’ decisions on whether and how to launder the proceeds of crime. One limitation of this article’s data is the lack of financial details across all three studies. While some respondents provided an accounting of their businesses, the data were not widely and consistently collected.

Nonetheless, we have elucidated three questions: How does the position in an illicit entrepreneurial career impact one’s choice of money management strategies; how do illicit entrepreneurs use licit structures to launder their money; and, why and how do illicit entrepreneurs innovate to launder the proceeds of their crimes? In answering these questions, this study shows that while illicit drug money is laundered through various strategies, these strategies are comparable across jurisdictions. Importantly, our study indicates that successful, small illicit entrepreneurs, that exist outside the context of large, hierarchical drug trafficking organizations, often do not indefinitely perpetuate their illicit enterprise; instead, they seek means to reduce their offending or to exit altogether. Thus, what may appear, on the surface, as laundering of criminal assets, may be, on closer inspection, a concerted long-term effort to go legal and desist from crime.

We confirm previous research findings that show that the volume of money generated by an illicit enterprise determines the money disposal strategies chosen (Malm and Bichler 2013; Naylor 2002). Small, illicit entrepreneurs with modest proceeds of crime most commonly engage in passive self-laundering strategies whereby they simply spend their money. Since retail drug distribution is the most common role in the illicit drug marketplace and, on aggregate, accounts for most of the markup taken (UNDCP 1998), passive self-laundering likely accounts for at least half of all dirty money that is laundered. However, combatting passive self-laundering is not cost effective; neither is identifying the provenance of income. Consequently, for many, a straightforward strategy to launder illicit revenue is to report it as taxable income.

Our findings illustrate that successful criminal entrepreneurs recognize when illicit income requires greater management, and this amount appears to be usually some tens of thousands of dollars or pounds of annual profits. Moreover, criminal entrepreneurs intuitively understand their options to launder money “good enough” to be spendable – opportunistic laundering through social networks, self-laundering through the tax-system, and direct investment into licit business – and the capacity limitations of those options. The study found that illicit enterprises restrict their size because increasing operational scale increases not only risks and complexity, but also financial costs. Finally, illicit entrepreneurs demonstrate the ability to establish alternative laundering routes, particularly when cash transfer is not an option.

To identify, investigate, and successfully prosecute the forms of passive and active laundering detailed in this article would require significant resources from both the police and tax authorities. “Following the money” is an expensive endeavor and may not be readily justified when the proceeds of crime are modest. Successful investigations often require either the development of well-placed informants who can accurately identify these illicit businesses and transactions and/or banks and non-bank institutions filing accurate suspicious transaction reports. Tax authorities in the U.S. and U.K. require that revenues be reported accurately; however, they do not audit the provenance of reported revenue. Resources for such investigations are virtually non-existent in either country. Policy strategies to curb the laundering strategies described may only increase overall market costs for licit consumers without reducing laundering transactions, given illicit entrepreneurs’ capacity to innovate when necessary. This is a historic and ongoing problem with money laundering regulation (Levi 2002).

## Transitions out of crime

The study found that those who engaged with the tax system seemed to transition away from crime by increasingly investing in their licit business interests while decreasing their illicit business activity, while those who found their illicit business activities to be too risky over time exited in a somewhat abrupt fashion. In both cases, the transition to leaving is often triggered by turning points that cause individuals to become more risk adverse and is manifested by obtaining licit, stable jobs or founding licit business ventures. Investing in the legitimate economy gave the offenders in this study a stake in conformity whether or not they had originally intended to exit the drug trade. Additionally, these criminal profits were not transferred offshore, nor were they amassed and hoarded in discreet locations, nor were they spent completely in the illicit economy; instead, they remained active in the local economy.

Encouraging transition from illicit to licit business is a strategy deployed in the developing world to limit carceral responses to behaviors driven by actors’ efforts to improve their economic station (Lupu 2004). Our data suggest that such “alternative development” strategies may be appropriate for Western contexts as well, especially as the legal status of drugs changes and as illicit entrepreneurs face barriers to starting a licit business, given capital deficits in the first instance and criminal records in the second. The findings of this study also indicate that legalization of previously illegal substances (e.g., cannabis) could assimilate illicit drug entrepreneurs into the formal economy, allowing them to trade regardless of drug-related criminal convictions. Such measures would be a continuation of many illicit entrepreneurs’ natural progression while providing an opportunity for these illicit entrepreneurs to contribute positively to society and the state’s coffers. Finally, this approach should not be confined to legalization scenarios; leniency should be afforded also to leavers who demonstrate a commitment to running a licit business.

## Declarations

The studies that form this article were approved by the university ethics committees at which the research was conducted. Participants included provided inform consent to participate.
